# Regulation of *Candida albicans* Interaction with Macrophages through the Activation of HOG Pathway by Genistein

**DOI:** 10.3390/molecules21020162

**Published:** 2016-01-28

**Authors:** Shuna Cui, Rabeay Y. A. Hassan, Anna Heintz-Buschart, Ursula Bilitewski

**Affiliations:** 1 Jiangsu Key Laboratory of Integrated Traditional Chinese and Western Medicine for Prevention and Treatment of Senile Diseases, Medical College of Yangzhou University, Huaihai Road 11, 225001 Yangzhou, China; sncui@yzu.edu.cn; 2 Helmholtz Centre for Infection Research (HZI), Inhoffenstr. 7, 38124 Braunschweig, Germany; rapy78@yahoo.com (R.Y.A.H.); anna.buschart@uni.lu (A.H.-B.); 3 Microanalysis Lab, Applied Organic Chemistry Department, National Research Centre (NRC), El Bohouthst., Dokki, 12622 Giza, Egypt; 4 Eco-Systems Biology Research Group, Luxembourg Centre for Systems Biomedicine, Campus Belval, 7 Avenue des Hauts-Fourneaux, L-4362 Esch-sur-Alzette, Luxembourg

**Keywords:** genistein, *Candida albicans*, HOG pathway, pathogen-macrophage interaction

## Abstract

The severity of infections caused by *Candida albicans*, the most common opportunistic human fungal pathogen, needs rapid and effective antifungal treatments. One of the effective ways is to control the virulence factors of the pathogen. Therefore, the current study examined the effects of genistein, a natural isoflavone present in soybeans, on *C. albicans*. The genistein-treated *C. albicans* cells were then exposed to macrophages. Although no inhibition effect on the growth rates of *C. albicans* was noted an enhancement of the immune response to macrophages has been observed, indicated by phagocytosis and release of cytokines TNF-α and IL-10. The effect of genistein on the enhanced phagocytosis can be mimicked by the fungicides fludioxonil or iprodione, which inhibit the histidine kinase Cos1p and lead to activation of HOG pathway. The western blot results showed a clear phosphorylation of Hog1p in the wild type strain of *C. albicans* after incubation with genistein. In addition, effects of genistein on the phosphorylation of Hog1p in the histidine kinase mutants *Δcos1* and *Δsln1* were also observed. Our results thus indicate a new bio-activity of genistein on *C. albicans* by activation of the HOG pathway of the human pathogen *C. albicans*.

## 1. Introduction

*Candida albicans* is an opportunistic pathogen, which colonizes almost 80% of the human population without causing disease. However, for immunocompromised patients, it has become the most frequent human fungal pathogen. *C. albicans* infections can range from superficial local infections to life-threatening systemic ones [1]. In healthy people *C. albicans* infections are controlled by the activities of both the innate and the adaptive immune systems [2]. Cells of the innate immune system, such as macrophages and polymorphonuclear leukocytes, possess a variety of pattern recognition receptors (PRRs) to recognize pathogenic fungi. The most prominent are toll-like receptors, of which TLR2 recognizes phospholipomannans, and C-type lectins, of which dectin-1 interacts with β-glucans from the fungal cell wall [3]. Binding of the fungus to these receptors leads to elimination of the fungus through phagocytosis and to the secretion of cytokines, which regulate the activity of other immune cells. Among the relevant cytokines are tumor necrosis factor (TNF-α) and interleukin-10 (IL-10). In live *C. albicans* yeast cells β-glucans are covered by a mannan layer, whereas heat-killing enhances β-glucan accessibility. Accordingly, the immune response is enhanced when heat-killed *C. albicans* are used instead of live *C. albicans*. In particular release of TNF-α and IL-10 was shown to depend on dectin-1 [3].

Classical antimycotic agents, which mainly target the structure or assembly of either the cell membrane or the cell wall, are challenged by increasing numbers of resistant strains and by severe side effects [4]. Thus, new effective therapeutic approaches are urgently needed. Among the recent treatment strategies which are discussed, is on the one hand the inhibition of virulence of the microorganism instead of its growth, and on the other hand the application of immune-modulating therapies [5,6].

Major contributions to virulence of *C. albicans* are its remarkable capabilities to form hyphae which allow penetration through the host cell layers, to adapt to various environmental niches in the host and to survive the immune system attack. Signal transduction pathways mediated by mitogen-activated protein kinases (MAPKs) play important roles in sensing and responding to changes in the environment. One of the most important stress response pathways of *C. albicans* is the high osmotic glycerol (HOG) pathway, which has been shown to mediate the response to osmotic, oxidative and temperature stress, to antifungal drugs which interfere with cell wall biosynthesis (adherence to host cells), and is involved in morphological changes and in the virulence of the organism [7].

In *C. albicans*, the HOG pathway is activated by the histidine kinases *Ca*Sln1p and *Ca*Nik1p (Cos1p) [8]. Under non-stress conditions, *Ca*Sln1p is active, which leads to its autophosphorylation and to the phosphorylation of the histidine phosphotransfer protein Ypd1p. The phosphate is then transferred to the response regulator *Ca*Ssk1p. Under hyperosmotic stress *Ca*Sln1p is inhibited, and neither Ypd1p nor Ssk1p are phosphorylated. Non-phosphorylated Ssk1p interacts with the MAPK3 Ssk2p, leading to its activation. This activation leads to phosphorylation of the MAPK2 Pbs2p and of the MAPK Hog1p. A non-functional HOG cascade leads to an increased sensitivity to osmotic and oxidative stress and as a consequence, to a decreased survival in the presence of phagocytes (neutrophils and macrophages) [9]. The *Δhog1* mutant shows significantly reduced virulence, it is more sensitive to immune cells [10]. Different chemical classes of fungicides were shown to target this pathway, such as phenylpyrroles (fludioxonil), dicarboximides (iprodione) and ambruticins. They trigger the phosphorylation of Hog1p by targeting the histidine kinase Cos1p.

It is nowadays realized that food constituents have a large effect on health, such as on the risks to develop metabolic disorders and other chronic diseases and even on the susceptibility to infectious diseases [11]. The origin of these effects frequently is a modulation of the activity of the immune system by plant constituents, in particular by polyphenols such as flavonoids. Among a great variety of natural flavonoids, genistein is one of the best studied. The isoflavone genistein was originally isolated from fermentation broth of a *Pseudomonas* sp. [12] and was later found in legumes, particularly in soybeans. It shows a variety of biological activities, for example as a phytoestrogen, an antioxidant, and as an inhibitor of a broad range of tyrosine kinases [13,14], and of the histidine kinase Sln1 in the yeast *S. cerevisiae* [15]. This leads to its chemoprotectant activities against cancers, cardiovascular disease and chronic inflammatory disorders [16,17,18,19,20]. However, the influence of genistein on *C. albicans* and on microbial pathogen–host interactions is not well understood. Therefore, the present study was designed to elucidate the implication of genistein on *C. albicans* and the interaction with immune cells.

## 2. Results

### 2.1. Genistein Treatment of C. albicans Enhances the Activity of Macrophages

Previously we had reported that extended treatment of macrophages with genistein inhibited the immune response to infections by *C. albicans*, as we had observed decreased phagocytic activity and TNF-α release when macrophages were incubated for at least 24 h with genistein [21]. This correlated well with the frequently reported inhibitory effect of genistein on TNF-α production by LPS (lipopolysaccharide)–stimulated macrophages [22].

**Figure 1:**
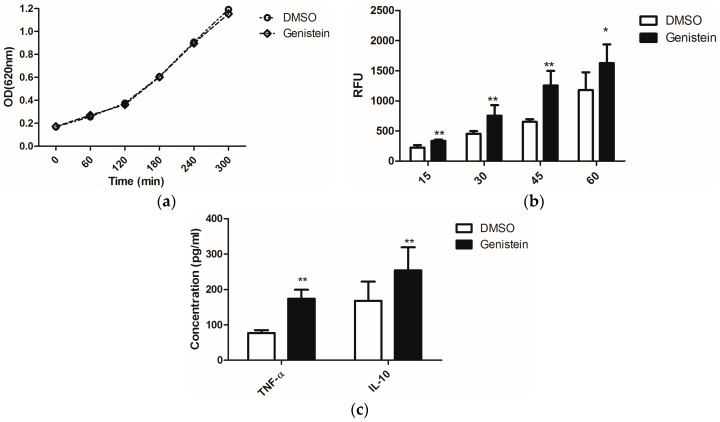
(**a**) Effects of genistein on growth of *C. albicans*. The optical density of the suspension was measured at 620 nm each hour using a μQuant microtiter plate reader; (**b**) Phagocytosis of genistein treated *C. albicans* by the murine macrophage cell line RAW 264.7. RFU: relative fluorescence units. ** *p* < 0.01, * *p* < 0.05; (**c**) Release of TNF-α and IL-10 by macrophages, which were incubated with genistein treated *C. albicans*; ** *p* < 0.01 (comparison with DMSO control).

On the other hand, enhanced phagocytosis efficiency of macrophages was reported when *C. albicans* was treated with sublethal concentrations of the antimycotic caspofungin, as this uncovered the glucans from the fungal cell wall and thus stimulated immune recognition via the PRR dectin-1 [23]. Due to the possible effects of genistein on fungal signal transduction cascades, which might influence as secondary effects also the cell wall structure, we investigated whether genistein could have similar effects as caspofungin. We treated *C. albicans* overnight with genistein and analysed the response of murine macrophages. The chosen genistein concentration of 100 μM had no influence on the growth of *C. albicans* (Figure 1a).

The phagocytic efficiency of the macrophages for the genistein-treated *C. albicans* was determined. In Figure 1b, the time course of the internalization is shown. The level of phagocytosis was significantly enhanced after infection with *C. albicans* for 15 min. and this effect lasted during the whole experimental period of 60 min.

From the enhanced phagocytosis of genistein–treated *C. albicans* we concluded that receptor–ligand interactions between *C. albicans* and macrophages were changed due to genistein treatment of the pathogen. As cytokine release is also activated by receptor–ligand interactions, such as dectin-1–β-glucan interactions, we determined the concentrations of TNF-α and IL-10 in the cell culture supernatants, as these cytokines are representative for cytokines, which are regulated by dectin-1 activation, though different signal transduction pathways may be involved [3]. In Figure 1c, data are shown for the production of TNF-α and IL-10, and a significant enhancement resulting from genistein treatment of the pathogen was observed (*p* < 0.01).

### 2.2. Genistein Treatment of C. albicans Activates the HOG Pathway of C. albicans

**Figure 2:**
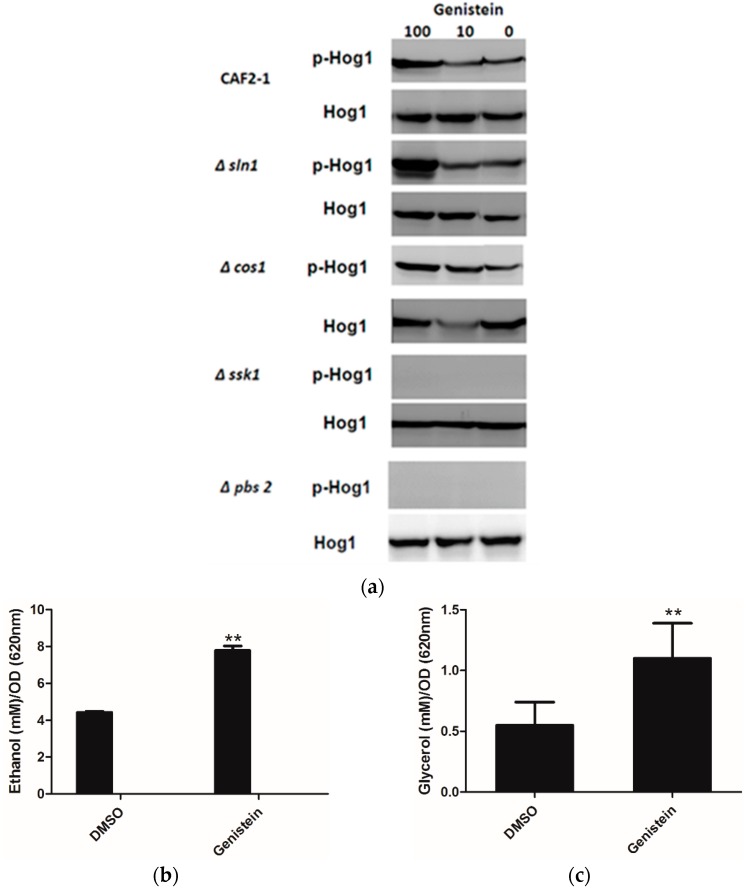
(**a**) Phosphorylation of Hog1p of *C. albicans* strains after treatment with genistein and solvent for 15min. Hog1p was detected by Hog1 (y-215) sc 9079 rabbit polyclonal IgG and phosphorylated Hog1p (Hog1-P) by Phospho-p38 MAPK (Thr180/182) 3D7 rabbit mAb at a site corresponding to 50 kDa; (**b**,**c**) Effect of genistein on glycerol and ethanol production in *C. albicans*. ** *p* < 0.01 (comparison with DMSO control).

As Huang reported an inhibitory effect of genistein on Sln1p of *S. cerevisiae* [15], and inhibition of Sln1p leads to the activation of the HOG pathway, we determined whether genistein activated the HOG pathway in *C. albicans*. The western blot results clearly showed phosphorylation of Hog1p in the wild type strain of *C. albicans* after incubation with genistein for 15 min. In addition, phosphorylation of Hog1p became phosphorylated, when the histidine kinase deletion mutants *Δcos1* and *Δsln1* were treated with genistein. However, in the MAPK2 mutant *Δpbs2* and in the mutant of the response regulator *Δssk1* the phosphorylation of Hog1p was not observed (Figure 2a).

Usually activation of the HOG pathway leads to increased glycerol production to protect yeast cells from osmotic stress. Since the HOG pathway was activated in *C. albicans* after treatment with genistein, glycerol concentrations were determined in the supernatants of *C. albicans* cultures. Increased glycerol concentrations were observed after 4 hour treatment (Figure 2b,c). However, also an increase in ethanol production was found, which could point to the utilization of fermentative metabolic pathways by *C. albicans* due to an inhibitory effect of genistein on the respiratory chain.

### 2.3. Enhanced Phagocytosis of Genistein Treated C. albicans Requires an Intact HOG Pathway

**Figure 3:**
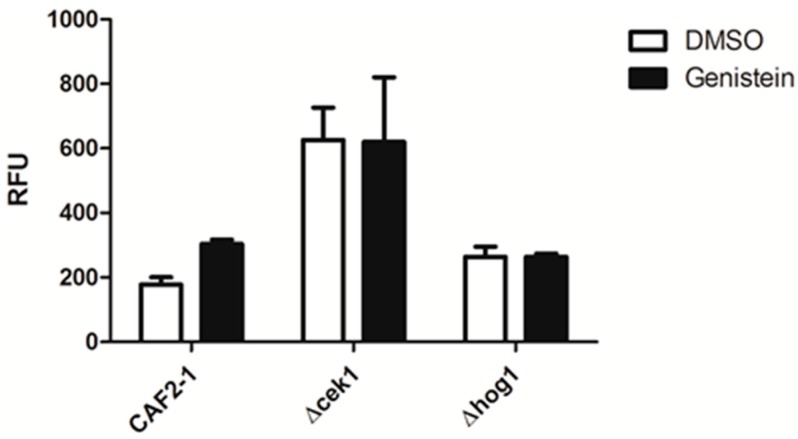
Phagocytosis of genistein treated mutants by RAW 264.7 macrophages in comparison to the reference strain treated CAF2-1. The phagocytosis time was 45 min. RFU: relative fluorescence units.

As genistein treatment of *C. albicans* activated the HOG pathway and as there is a complex relationship between the MAP kinases of *C. albicans*, we analyzed the relevance of the MAP kinases Hog1p and Cek1p for the enhanced phagocytic activity of macrophages for genistein treated *C. albicans* by using the respective single gene deletion mutants. The result showed that the phagocytosis efficiency for *Δcek1* and *Δhog1* was not affected by genistein treatment, suggesting that these MAP kinases were essential for the genistein effect on the phagocytosis efficiency of the wild type strain (Figure 3).

### 2.4. Fludioxonil and iprodione Treated C. albicans are More Susceptible to Macrophage

Fludioxonil and iprodione are well known fungicides which are used in agriculture against fungal plant pathogens. They target fungal type III histidine kinases. The *C. albicans* histidine kinase *Ca*Nik1p (Cos1p) is also a type III histidine kinase and we had shown that it is the target of iprodione and fludioxonil [24,25]. Treatment of *C. albicans* with these compounds did not significantly affect growth (data not shown), but lead to activation of Hog1p and induce glycerol production in *C. albicans* [24,25].

**Figure 4:**
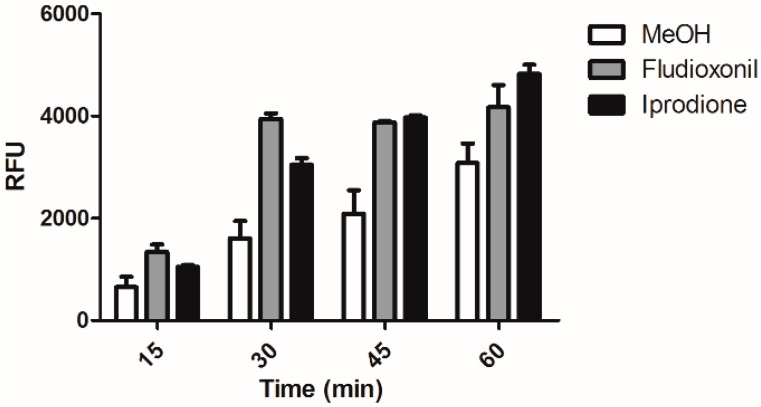
Phagocytosis of fludioxonil and iprodione treated *C. albicans* by the murine macrophage cell line RAW 264.7. RFU: relative fluorescence units.

As we observed that treatment of *C. albicans* by macrophage and led to phosphorylation of Hog1p and that Hog1p was essential for the enhanced phagocytosis of genistein treated *C. albicans*, we speculated whether treatment of *C. albicans* with either fludioxonil or iprodione would also lead to the enhanced phagocytosis. As shown in Figure 4 also treatment of *C. albicans* with fludioxonil or iprodione led to significantly enhanced levels of phagocytosis during the whole experimental period of 60 min. The result showed the same trend as after genistein treatment.

## 3. Discussion

In the current study, genistein effects on the biological activities of *C. albicans* as well as on the *C. albicans–*macrophage interactions have been investigated. The growth rates of *C. albicans*, either in rich YPD medium or the buffered RPMI1640 medium, was not affected by genistein. The HOG pathway of *C. albicans* was activated in presence of genistein, this was confirmed by the western blot (phosphorylation of p38) and by detection the extracellular accumulation of glycerol.

The effects on the biological activities of *C. albicans*, and HOG pathway, may play a role on the virulence capability of the pathogen organism. From an enhancement level of phagocytosis and also the stimulated production of the pro-inflammatory cytokine TNF-α and of the anti-inflammatory cytokine IL-10, we could confirm that genistein could influence the pathogenesis of *C. albicans*. Releasing TNF-α can at early stages of infection has been reported to have a positive feedback on phagocytosis [26]. Therefore, the increased secretion of TNF-α may be the reason for the enhanced phagocytosis of *C. albicans*.

The histidine kinase Sln1p of *S. cerevisiae* was reported to be inhibited by genistein [15] and increased glycerol production is a well-established response to Sln1p inhibition due to osmotic stress. Moreover, genistein has been found to regulate the molecules in the MAPK pathway in different ways: genistein inhibits TGF-β-mediated p38 MAP kinase activation and matrix metalloproteinase type 2 [27]. It has been found to be effective in preventing cytokine-induced ERK1/2 activation and promoted apoptotic cell death [28]. On the other hand, genistein also could potentiate the phosphorylation of p38, ERK1/2 in breast cancer lines and macrophage [21,29,30,31]. Therefore, we speculated that genistein could activate the HOG pathway in *C. albicans*, which is further proved by western blot results. Genistein activates the HOG pathway by phosphorylation of Hog1 after 15 min. This activation is through Ssk1p and Pbs2p, but not due to the inhibition of histidine kinase Sln1p and Cos1p. Moreover, genistein stimulated glycerol and ethanol synthesis in *C. albicans*; phagocytosis efficiency for *Δcek1* and *Δhog1* was not affected by genistein treatment, suggesting that the genistein effect of the wild type could be related to the pathways. Those results for the first time showed that genistein activates the HOG pathway in *C. albicans*.

Furthermore, in comparison with fludioxonil and iprodione, we found those inhibitors treated *C. albicans* show the similar effect like genistein treated *C. albicans*. For example, fludioxonil and iprodione treated *C. albicans* cells are more susceptible to macrophage.

In conclusion, our results show for the first time that genistein activates the HOG pathway of *C. albicans* and enhances the immune responses, using phagocytosis and cytokines production as indicators. Our results highlight a new effect of genistein on the human pathogen *C. albicans* and point out new pharmacological activities of genistein and alternative strategies for immunostimulation in host-pathogen system by treatment of the pathogen instead of the immune cells.

## 4. Experimental Section

### 4.1. Materials

Genistein was purchased from Sigma (St. Louis, MO, USA); a stock solution of genistein at a concentration of 200 mM was prepared in dimethyl sulfoxide (DMSO, Biomol GmbH, Hamburg, Germany), the working concentration was 10 mM diluted from stock solution by DMSO and frozen at −20 °C. Fludioxonil and iprodione was purchased from Fluka (Hamburg, Germany), 10 mg/mL stock solution was prepared in methanol. Dulbecco’s modified Eagle’s Medium (DMEM), fetal bovine serum (FBS), and RPMI 1640 media with l-glutamine were from Lonza (Verviers, Belgium). MOPS were purchased from Geyer (Hamburg, Germany). Glycerol dehydrogenase (GDH) (1 KU) from *cellulomonas* sp. was purchased from Sigma and 1 KU was solved in 3.33 mL MQ water. Nicotinadenine dinucleotide (NAD^+^) was from Biomol (Hamburg, Germany). Fluorescein isothiocyanate (FITC) was from Sigma and a stock solution (100 mg/mL) was prepared in DMSO. Phospho p-38 MAPK (Thr180/182) 3D7 rabbit mAb together with HRP-linked anti-rabbit IgG antibody were from Cell Signaling Technology (Danvers, MA, USA). Hog1 (y-215) sc 9079 rabbit polyclonal IgG was from Santa Cruz Biotechnology (Dallas, TX, USA).

### 4.2. Strains and Culture Condition

The test organism *C. albicans* strains (Table 1) were grown overnight in YPD broth (Sigma) at 30 °C on a shaker. The yeast suspensions were diluted to an OD_620nm_ (optical density) of 0.2 (determined with a sample volume of 180 μL with the μQuant microtiter plate reader (BioTek Instruments GmbH, Bad Friedrichshall, Germany) in YPD medium or in RPMI 1640 medium (with 165 mM MOPS and 0.2% glucose pH 7.5) and allowed to grow for 2–3 h.

**Table 1:** *C. albicans* strains used in this study.

Strains	Synonym	Genotype	Reference
CAF2-1		*∆ura3*::imm434/URA3	[32]
∆*casln1*	CaSLN1	∆*ura3*::imm434/∆*ura3*::imm434 *∆casln1*::hisG/*∆casln1*/hisG-URA3-hisG	[8]
∆*cos1*	LAC17	∆*ura3*::imm434/∆*ura3*::imm434 *∆cos1*::hisG/*∆cos1*/hisG-URA3-hisG	[8]
∆*pbs2*	BRD3	∆*ura3::imm434/*∆*ura3::imm434* ∆*his1::hisG/*∆*his1::hisG*∆*pbs2::cat/*∆*pbs2::cat-URA3-cat*	[33]
∆*ssk1*	CSSK21	∆*ura3*::imm434/∆*ura3*::imm434 *∆cassk1*::hisG/*∆cassk1*/hisG-URA3-hisG	[34]
∆*cek1*	CK43B-16	*ura3/ura3∆cek1::hisG/∆cek1::hisG-URA3-hisG*	[35]
∆*hog1*	CNC13	*hog1::hisG/hog1::hisG-*URA3*-hisG ∆his1*::hisG/∆*his1*::hisG	[36]

The murine macrophage cell line RAW 264.7 was purchased from the American Type Culture Collection (ATCC, Rockville, MD, USA). The cells were grown in DMEM supplemented with 10% fetal bovine serum (FBS) and 100 U/mL penicillin and 100 μg/mL streptomycin at 37 °C in a 5% CO_2_ air atmosphere. The human epithelial carcinoma cell line A431 cell was also purchased from ATCC. The cells were grown in RPMI 1640 media with L-glutamine supplemented with 10% FBS at 37 °C in a 5% CO_2_ air atmosphere.

### 4.3. Preparation of Candida albicans for Phagocytosis Assay

*C. albicans* CAF2-1 pre-culture was diluted to OD = 0.1 in 20 mL YPD medium supplemented or not with genistein (the final concentration was 100 μM) and fludioxonil (20 μg/mL) and iprodione (20 μg/mL) were grown overnight at 30 °C with orbital shaking at 160 rpm. For fluorescence-labeling, 1 × 10^8^ yeasts were harvested by centrifugation (13,000 rpm, 5 min, 24 °C), washed twice in 1 mL PBS and stained overnight with 1 mL of 500 μg/mL FITC at 4 °C. Yeasts were washed three times in PBS to remove excessive dye before use. Even though we always followed the same staining protocol for the yeasts we observed deviations in staining efficiency depending on the storage time of the fluorescent dye (FITC).

### 4.4. Phagocytosis Assay

Phagocytosis of *C. albicans* by macrophages was quantified as described previously [37]. Briefly: 100 μL of 2 × 10^6^ macrophages/mL were seeded in each well of 96 well microtiter plates (Nunc, Darmstadt, Germany) followed by incubation for 2 h to let the cells adhere to the plates. The supernatant was removed from the macrophage cell culture and 100 μL of the yeast suspension in medium were added. Phagocytosis was allowed to proceed at 37 °C in 5% CO_2_ for different periods of time. The medium was removed and 100 μL trypan blue (250 μL/mL in PBS) were added to quench the fluorescence of yeasts which were not internalized. After an incubation of 1 min at room temperature, the trypan blue solution was removed. The number of internalized yeasts was estimated from fluorescence measurements (λ_Ex_ 480 nm and λ_Em_ 520 nm) through the bottom of the plates by a fluorescence multi-well plate reader (Syngery 4^®^ BioTek Instruments GmbH). In our diagrams we show the real data from the fluorescence multi-well plate reader. They are influenced by the staining efficiency achieved in the respective batch (see Section 4.3), but also by the chosen sensitivity of the photomultiplier of the reader. Thus, these absolute data can be compared in a series of experiments. However, for comparison between series, normalization would be required. In our diagrams we show a representative example for the described results. 

### 4.5. Cytokine Determination

TNF-α and IL-10 were determined following previously published protocols [21]. 3 × 10^5^ macrophages/mL (100 μL) were seeded in each well of 96 well microtiter plates and allowed to adhere for 2 h. 3 × 10^6^ *C. albicans*/mL were harvested from an overnight culture with or without genistein by centrifugation (13,000 rpm, 5 min, 24 °C), and washed twice with 1 mL PBS. They were suspended in DMEM cell culture medium. The supernatant was removed from the macrophage cell culture and 100 μL of the yeast suspension were added. The ratio of macrophages: yeast was 1:10. TNF-α concentrations were determined after a yeast-macrophage incubation time of 1 h, IL-10 concentrations were determined after 5 h. The concentrations of IL-10 and TNF-α were quantified by ELISA (eBioscience, Inc., San Diego, CA, USA) according to the instructions given by the manufacturer, only that half area high binding 96 well microtiter plates (Greiner Bio-One GmbH, Frickenhausen, Germany) were used, so that volumes of antibody solutions and samples were reduced to half of the amounts given by the kit manufacturer. The results are expressed in pg/mL.

### 4.6. Protein Analysis

Western blot for phosphorylation of Hog1 after genistein treatment was detected according to Buschart *et al.* [25]. Briefly, *C. albicans* strains were grown as described above. The culture was diluted to OD_620nm_ 0.2, and subjected to treatments with or without genistein for 15 or 30 min. Cells were harvested by centrifugation at room temperature and frozen in liquid nitrogen. Frozen cell pellets were disrupted with a Mini-Dismembrator U (B. Braun Biotech, Melsungen, Germany) in the presence of lysis buffer (10 mM sodium phosphate buffer pH 8.5, supplemented with 5 mM NaCl, 5 mM KCl, 11 g/L glucose, protease and phosphatase inhibitors (complete, mini and PhosSTOP, Roche, Wurmisweg, Switzerland). After centrifugation at 10,000× *g*, the supernatant are collected. Protein concentration of the supernatant was determined by BCA methods.

Twenty μg of protein was separated by SDS-PAGE (12.5%), proteins were blotted and phosphorylated Hog1 was detected using phospho p-38 MAPK (Thr180/182) 3D7 rabbit mAb (Cell Signaling Technology) together with HRP-linked anti-rabbit IgG antibody (Cell Signaling Technology). The anti-phospho p-38 MAPK antibody had to be used, as there are only some antibodies available, which are specific for proteins from *C. albicans.* However, Hog1p is the *Candida*–homologue to the mammalian MAPK p-38 and the relevant epitopes of the phosphorylated proteins are so similar that they are recognized by the same antibody.

The membrane was stripped and total Hog1 was detected using Hog1 (y-215) sc 9079 rabbit polyclonal IgG (Santa Cruz Biotechnology) and HRP-linked anti-rabbit antibody, and visualized using chemiluminescence (ECL Advance Western Blotting Detection Kit, GE Healthcare, Freiburg, Germany). At least three independent experiments were conducted and data from one representative experiment is shown.

### 4.7. Ethanol and Glycerol Determination

Both glycerol and ethanol concentrations were determined by enzymatic assays based on glycerol dehydrogenase (GDH) and ethanol dehydrogenase, respectively [38]. Quantification in supernatants was based on the photometric determination of NADH at 340 nm, and the assays were performed in volume reduced 96-well transparent microtiter plates (Corning^®^, Corning, NY, USA).

### 4.8. Statistical Analysis

Statistical significances were determined by Student’s *t*-test and statistical significance was assumed by *p* < 0.05.

## Abbreviations

The following abbreviations are used in this manuscript:

MAPKs: mitogen-activated protein kinases
HOG: high osmotic glycerol
RFU: relative fluorescence units

## References

[1] Calera J.A., Calderone R. (1999). Histidine kinase, two-component signal transduction proteins of *Candida albicans* and the pathogenesis of candidosis. Mycoses.

[2] Romani L. (2000). Innate and adaptive immunity in *Candida albicans* infections and saprophytism. J. Leukoc. Biol..

[3] Gow N.A., Netea M.G., Munro C.A., Ferwerda G., Bates S., Mora-Montes H.M., Walker L., Jansen T., Jacobs L., Tsoni V. (2007). Immune recognition of *Candida albicans* beta-glucan by dectin-1. J. Infect. Dis..

[4] Kruppa M., Goins T., Cutler J.E., Lowman D., Williams D., Chauhan N., Menon V., Singh P., Li D., Calderone R. (2003). The role of the *Candida albicans* histidine kinase [CHK1) gene in the regulation of cell wall mannan and glucan biosynthesis. FEMS Yeast Res..

[5] Chauhan N., Calderone R. (2008). Two-component signal transduction proteins as potential drug targets in medically important fungi. Infect. Immunity.

[6] Casadevall A., Pirofski L.A. (2001). Adjunctive immune therapy for fungal infections. Clin. Infect. Dis..

[7] Kruppa M., Calderone R. (2006). Two-component signal transduction in human fungal pathogens. FEMS Yeast Res..

[8] Nagahashi S., Mio T., Ono N., Yamada-Okabe T., Arisawa M., Bussey H., Yamada-Okabe H. (1998). Isolation of CaSLN1 and CaNIK1, the genes for osmosensing histidine kinase homologues, from the pathogenic fungus *Candida albicans*. Microbiology.

[9] Alonso-Monge R., Carvaihlo S., Nombela C., Rial E., Pla J. (2009). The Hog1 MAP kinase controls respiratory metabolism in the fungal pathogen *Candida albicans*. Microbiology.

[10] Arana D.M., Alonso-Monge R., Du C., Calderone R., Pla J. (2007). Differential susceptibility of mitogen-activated protein kinase pathway mutants to oxidative-mediated killing by phagocytes in the fungal pathogen *Candida albicans*. Cell. Microb..

[11] World Health Organization (2003). Diet, Nutrition and the Prevention of Chronic Diseases.

[12] Ogawara H., Akiyama T., Ishida J., Watanabe S., Suzuki K. (1986). A specific inhibitor for tyrosine protein kinase from *Pseudomonas*. J. Antibiot..

[13] Akiyama T., Ishida J., Nakagawa S., Ogawara H., Watanabe S., Itoh N., Shibuya M., Fukami Y. (1987). Genistein, a specific inhibitor of tyrosine-specific protein kinases. J. Biol. Chem..

[14] Akiyama T., Ogawara H. (1991). Use and specificity of genistein as inhibitor of protein-tyrosine kinases. Methods Enzymol..

[15] Huang J., Nasr M., Kim Y., Matthews H.R. (1992). Genistein inhibits protein histidine kinase. J. Biol. Chem..

[16] Ruetten H., Thiemermann C. (1997). Effects of tyrphostins and genistein on the circulatory failure and organ dysfunction caused by endotoxin in the rat: A possible role for protein tyrosine kinase. Br. J. Pharmacol..

[17] Sasamura H., Takahashi A., Yuan J., Kitamura H., Masumori N., Miyao N., Itoh N., Tsukamoto T. (2004). Antiproliferative and antiangiogenic activities of genistein in human renal cell carcinoma. Urology.

[18] Sasamura H., Takahashi A., Miyao N., Yanase M., Masumori N., Kitamura H., Itoh N., Tsukamoto T. (2002). Inhibitory effect on expression of angiogenic factors by antiangiogenic agents in renal cell carcinoma. Br. J. Cancer.

[19] Li Y., Kucuk O., Hussain M., Abrams J., Cher M.L., Sarkar F.H. (2006). Antitumor and antimetastatic activities of docetaxel are enhanced by genistein through regulation of osteoprotegerin/receptor activator of nuclear factor-kappaB (RANK)/RANK ligand/MMP-9 signaling in prostate cancer. Cancer Res..

[20] Farina H.G., Pomies M., Alonso D.F., Gomez D.E. (2006). Antitumor and antiangiogenic activity of soy isoflavone genistein in mouse models of melanoma and breast cancer. Oncol. Rep..

[21] Cui S., Wienhoefer N., Bilitewski U. (2014). Genistein induces morphology change and G2/M cell cycle arrest by inducing p38 MAPK activation in macrophages. Int. Immunopharmacol..

[22] Choi C., Cho H., Park J., Cho C., Song Y. (2003). Suppressive effects of genistein on oxidative stress and NFkappaB activation in RAW 264.7 macrophages. Biosci. Biotechnol. Biochem..

[23] Batbayar S., Lee D.H., Kim H.W. (2012). Immunomodulation of fungal beta-Glucan in host defense signaling by dectin-1. Biomol. Ther..

[24] Buschart A., Burakowska A., Bilitewski U. (2012). The fungicide fludioxonil antagonizes fluconazole activity in the human fungal pathogen *Candida albicans*. J. Med. Microb..

[25] Buschart A., Gremmer K., El-Mowafy M., van den Heuvel J., Mueller P.P., Bilitewski U. (2012). A novel functional assay for fungal histidine kinases group III reveals the role of HAMP domains for fungicide sensitivity. J. Biotechnol..

[26] Murray R.Z., Kay J.G., Sangermani D.G., Stow J.L. (2005). A role for the phagosome in cytokine secretion. Science.

[27] Huang X., Chen S., Xu L., Liu Y., Deb D.K., Platanias L.C., Bergan R.C. (2005). Genistein inhibits p38 map kinase activation, matrix metalloproteinase type 2, and cell invasion in human prostate epithelial cells. Cancer Res..

[28] Liu H., Du J., Hu C., Qi H., Wang X., Wang S., Liu Q., Li Z. (2009). Delayed activation of extracellular-signal-regulated kinase 1/2 is involved in genistein- and equol-induced cell proliferation and estrogen-receptor-alpha-mediated transcription in MCF-7 breast cancer cells. J. Nutr. Biochem..

[29] Frey R.S., Singletary K.W. (2003). Genistein activates p38 mitogen-activated protein kinase, inactivates ERK1/ERK2 and decreases Cdc25C expression in immortalized human mammary epithelial cells. J. Nutr..

[30] Sanchez Y., Amran D., Fernandez C., de Blas E., Aller P. (2008). Genistein selectively potentiates arsenic trioxide-induced apoptosis in human leukemia cells via reactive oxygen species generation and activation of reactive oxygen species-inducible protein kinases (p38-MAPK, AMPK). Int. J. Cancer.

[31] Li Z., Li J., Mo B., Hu C., Liu H., Qi H., Wang X., Xu J. (2008). Genistein induces G2/M cell cycle arrest via stable activation of ERK1/2 pathway in MDA-MB-231 breast cancer cells. Cell Biol. Toxicol..

[32] Fonzi W.A., Irwin M.Y. (1993). Isogenic strain construction and gene mapping in *Candida albicans*. Genetics.

[33] Arana D.M., Nombela C., Alonso-Monge R., Pla J. (2005). The Pbs2 MAP kinase kinase is essential for the oxidative-stress response in the fungal pathogen *Candida albicans*. Microbiology.

[34] Calera J.A., Zhao X.J., Calderone R. (2000). Defective hyphal development and avirulence caused by a deletion of the SSK1 response regulator gene in *Candida albicans*. Infect. Immunity.

[35] Navarro-Garcia F., Sanchez M., Pla J., Nombela C. (1995). Functional characterization of the MKC1 gene of *Candida albicans*, which encodes a mitogen-activated protein kinase homolog related to cell integrity. Mol. Cell Biol..

[36] San Jose C., Monge R.A., Perez-Diaz R., Pla J., Nombela C. (1996). The mitogen-activated protein kinase homolog HOG1 gene controls glycerol accumulation in the pathogenic fungus *Candida albicans*. J. Bacterial..

[37] Klippel N., Bilitewski U. (2007). Phagocytosis assay based on living *Candida albicans* for the detection of effects of chemicals on macrophage function. Anal. Lett..

[38] Wesolowski J., Hassan R.Y., Reinhardt K., Hodde S., Bilitewski U. (2010). Antifungal compounds redirect metabolic pathways in yeasts: Metabolites as indicators of modes of action. J. Appl. Microbial..

